# Supplementary material for: Biological invasions alter environmental microbiomes: A meta-analysis
Source: PLoS One. 2020 Oct 22;15(10):e0240996. doi: 10.1371/journal.pone.0240996 (PMC7580985; doi:10.1371/journal.pone.0240996)
Supplement: S1 Fig — From: Moher D, Liberati A, Tetzlaff J, Altman DG, The PRISMA Group (2009). Preferred Reporting Items for Systematic Reviews and Meta-Analyses: The PRISMA Statement. PLoS Med 6(7): e1000097. doi:10.1371/journal.pmed1000097. (PDF) [file pone.0240996.s001.pdf]

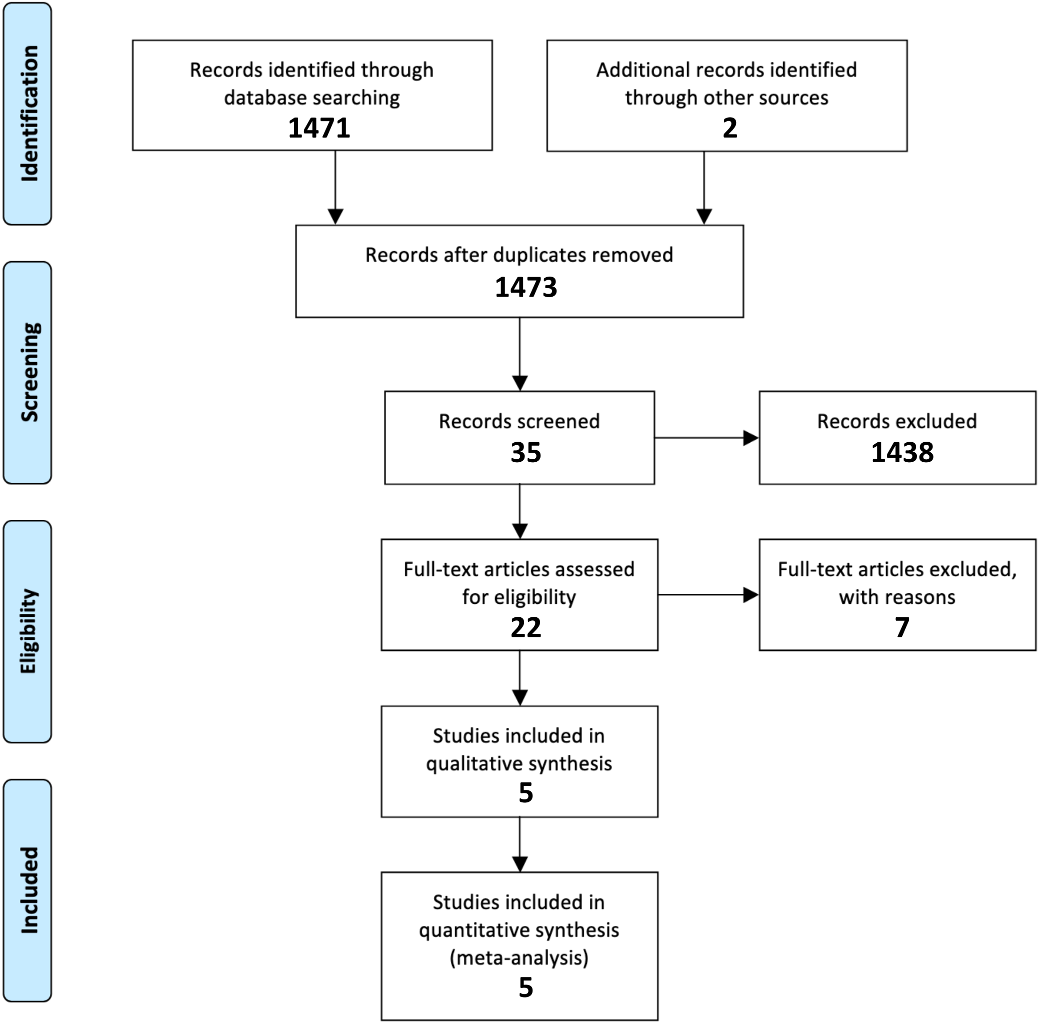
Identification
Screening
Eligibility
Included
Records identified through database searching
1471
Additional records identified through other sources
2
Records after duplicates removed
1473
Records screened
35
Records excluded
1438
Full-text articles assessed for eligibility
22
Full-text articles excluded, with reasons
7
Studies included in qualitative synthesis
5
Studies included in quantitative synthesis (meta-analysis)
5
