# Supplementary material for: Biological invasions alter environmental microbiomes: A meta-analysis
Source: PLoS One. 2020 Oct 22;15(10):e0240996. doi: 10.1371/journal.pone.0240996 (PMC7580985; doi:10.1371/journal.pone.0240996)
Supplement: S2 Table — In Model 1 we included studyID as random factor, while in Model 2 we included both studyID and environment (soil or water) as random factors. (PDF) [file pone.0240996.s003.pdf]

Biological invasions alter environmental microbiomes: a meta-analysis

Antonino Malacrinò, Victoria A. Sadowski, Tvisha K. Martin, Nathalia Cavichiolli de Oliveira, Ian J. Brackett, James D. Feller, Kristian J. Harris, Orlando Combita Heredia, Rosa Vescio, Alison E. Bennett

S2 Table. Comparison of two different linear mixed-effects models testing the effect of biological *sample type* (invaded or control), *organism* (plant, mammal, mussel), and their interactions, on Shannon diversity index of the environmental microbiome. In Model 1 we included *studyID* as random factor, while in Model 2 we included both *studyID* and *environment* (soil or water) as random factors.

**Model 1**, AIC = -160.88<br>**lmer(Shannon ~ Sample_type * Organism_group * (1|Study_ID))**			
	**χ^2**	**df**	**P**
Sample_type	3.85	1	0.04
Organism_group	84.18	2	<0.001
Sample_type Organism_ group*	3.51	2	0.17
**Model 2**, AIC = -158.88<br>**lmer(Shannon ~ Sample_type * Organism_group * (1|Study_ID) * (1|Environment))**			
	**χ^2**	**df**	**P**
Sample_type	3.85	1	0.04
Organism	59.7	2	<0.001
Sample_type Organism*	3.51	2	0.17
