# Supplementary material for: Biological invasions alter environmental microbiomes: A meta-analysis
Source: PLoS One. 2020 Oct 22;15(10):e0240996. doi: 10.1371/journal.pone.0240996 (PMC7580985; doi:10.1371/journal.pone.0240996)
Supplement: S3 Table — In Model 1 we included studyID and speciesID as stratification factor, while in Model 2 we included studyID, speciesID and environment (soil or water) as stratification factors. (PDF) [file pone.0240996.s004.pdf]

Biological invasions alter environmental microbiomes: a meta-analysis

Antonino Malacrinò, Victoria A. Sadowski, Tvisha K. Martin, Nathalia Cavichiolli de Oliveira, Ian J. Brackett, James D. Feller, Kristian J. Harris, Orlando Combita Heredia, Rosa Vescio, Alison E. Bennett

S3 Table. Comparison of two different PERMANOVA models testing the effect of biological *sample type* (invaded or control), *organism* (plant, mammal, mussel), and their interactions, on the structure of the environmental microbiome. In Model 1 we included *studyID* and *speciesID* as stratification factor, while in Model 2 we included *studyID*, *speciesID* and *environment* (soil or water) as stratification factors.

**Model 1**<br>**~ Sample_type * Organism_group, strata = c("Study_ID", "Species_ID")**			
	**R^2**	**F**	**P**
Sample_type	0.011	6.68	<0.001
Organism_group	0.411	118.86	<0.001
Sample_type Organism_group*	0.007	2.1	0.01
**Model 2**<br>**~ Sample_type * Organism_group, strata= c("Study_ID", “Environment”, "Species_ID")**			
	**R^2**	**F**	**P**
Sample_type	0.011	6.68	<0.001
Organism_group	0.411	118.86	<0.001
Sample_type Organism_group*	0.007	2.1	0.02
