# Supplementary material for: Biological invasions alter environmental microbiomes: A meta-analysis
Source: PLoS One. 2020 Oct 22;15(10):e0240996. doi: 10.1371/journal.pone.0240996 (PMC7580985; doi:10.1371/journal.pone.0240996)
Supplement: S4 Table — In Model 1 we included studyID as random factor, while in Model 2 we included both studyID and environment (soil or water) as random factors. (PDF) [file pone.0240996.s005.pdf]

Biological invasions alter environmental microbiomes: a meta-analysis

Antonino Malacrinò, Victoria A. Sadowski, Tvisha K. Martin, Nathalia Cavichiolli de Oliveira, Ian J. Brackett, James D. Feller, Kristian J. Harris, Orlando Combita Heredia, Rosa Vescio, Alison E. Bennett

S4 Table. Results from two different the linear mixed-effects model testing the abundance of each bacterial family against *sample type* (invaded or control), *organism* (plant, mammal, mussel), and their interactions. In Model 1 we included *studyID* as random factor, while in Model 2 we included both *studyID* and *environment* (soil or water) as random factors.

	**Model 1***		**Model 2§**	
**Family**	**χ^2**	**P**	**χ^2**	**P**
Burkholderiaceae	0.22	0.63	0.18	0.67
Sporichthyaceae	0.02	0.86	0.03	0.86
Chthoniobacteraceae	**17.28**	**<0.001**	**17.28**	**<0.001**
Chitinophagaceae	**27.77**	**<0.001**	**27.78**	**<0.001**
Gemmataceae	**15.04**	**<0.001**	**17.42**	**<0.001**
Solibacteraceae (Subgroup 3)	**30.75**	**<0.001**	**30.68**	**<0.001**
Gemmatimonadaceae	**86.45**	**<0.001**	**86.46**	**<0.001**
Xanthobacteraceae	1.23	0.26	1.22	0.27
Ord. Tepidisphaerales	3.80	0.05	3.76	0.05
Sphingomonadaceae	**14.20**	**<0.001**	**14.21**	**<0.001**
Pyrinomonadaceae	0.47	0.49	0.47	0.49
Pedosphaeraceae	**12.09**	**<0.001**	**12.10**	**<0.001**
Solirubrobacteraceae	**10.36**	**<0.01**	**10.27**	**<0.001**
Acetobacteraceae	**33.30**	**<0.001**	**33.30**	**<0.001**
Ord. Solirubrobacterales	2.74	0.09	2.71	0.10
Blastocatellaceae	**32.42**	**<0.001**	**32.43**	**<0.001**
Pirellulaceae	**41.78**	**<0.001**	**41.10**	**<0.001**
Beijerinckiaceae	4.01	0.05	4.01	0.05
Haliangiaceae	2.32	0.12	2.29	0.13
Micromonosporaceae	**6.17**	**0.01**	**6.12**	**0.01**
Nitrosomonadaceae	**5.67**	**0.02**	**5.69**	**0.02**

*lmer(Abundance ~ Sample_type * (1|Study_ID) * (1|Invasive_species))
§lmer(Abundance ~ Sample_type * (1|Study_ID) * (1|Environment) * (1|Invasive_species))
