# Supplementary material for: Biological invasions alter environmental microbiomes: A meta-analysis
Source: PLoS One. 2020 Oct 22;15(10):e0240996. doi: 10.1371/journal.pone.0240996 (PMC7580985; doi:10.1371/journal.pone.0240996)
Supplement: S5 Table — Differences are assessed using a linear mixed-effects model testing the normalized proportions of each bacterial family against sample type (Model 1 in S4 Table). (PDF) [file pone.0240996.s006.pdf]

Biological invasions alter environmental microbiomes: a meta-analysis

Antonino Malacrinò, Victoria A. Sadowski, Tvisha K. Martin, Nathalia Cavichiolli de Oliveira, Ian J. Brackett, James D. Feller, Kristian J. Harris, Orlando Combita Heredia, Rosa Vescio, Alison E. Bennett

S5 Table. Comparison of the relative proportion of each bacterial family between control and invaded environments. Differences are assessed using a linear mixed-effects model testing the normalized proportions of each bacterial family against *sample type* (Model 1 in S4 Table).

**Family**	**Control**		**Invaded**		χ^2	**P**
	Mean	*SE*	*Mean*	*SE*		
Acetobacteraceae	0.0115	0.0004	0.0090	0.0004	33.3	**<0.001**
Beijerinckiaceae	0.0128	0.0002	0.0125	0.0003	4.01	0.05
Blastocatellaceae	0.0131	0.0005	0.0135	0.0005	32.42	**<0.001**
Burkholderiaceae	0.0315	0.0022	0.0404	0.0029	0.22	0.63
Chitinophagaceae	0.0499	0.0012	0.0527	0.0012	27.77	**<0.001**
Chthoniobacteraceae	0.0457	0.0016	0.0420	0.0015	17.28	**<0.001**
Gemmataceae	0.0290	0.0008	0.0253	0.0007	15.04	**<0.001**
Gemmatimonadaceae	0.0373	0.0013	0.0316	0.0011	86.45	**<0.001**
Haliangiaceae	0.0130	0.0003	0.0118	0.0004	2.32	0.12
Micromonosporaceae	0.0135	0.0004	0.0118	0.0004	6.17	**0.01**
Nitrosomonadaceae	0.0118	0.0002	0.0116	0.0003	5.67	**0.02**
Ord. Solirubrobacterales	0.0118	0.0004	0.0111	0.0004	2.74	0.09
Ord. Tepidisphaerales	0.0250	0.0008	0.0231	0.0010	3.8	0.05
Pedosphaeraceae	0.0186	0.0005	0.0179	0.0004	12.09	**<0.001**
Pirellulaceae	0.0122	0.0003	0.0132	0.0004	41.78	**<0.001**
Pyrinomonadaceae	0.0211	0.0008	0.0197	0.0008	0.47	0.49
Solibacteraceae (Subgroup 3)	0.0239	0.0009	0.0189	0.0007	30.75	**<0.001**
Solirubrobacteraceae	0.0113	0.0003	0.0103	0.0003	10.36	**<0.01**
Sphingomonadaceae	0.0255	0.0008	0.0267	0.0007	14.2	**<0.001**
Sporichthyaceae	0.0073	0.0019	0.0154	0.0027	0.02	0.86
Xanthobacteraceae	0.0275	0.0008	0.0238	0.0009	1.23	0.26
