# Supplementary material for: Biological invasions alter environmental microbiomes: A meta-analysis
Source: PLoS One. 2020 Oct 22;15(10):e0240996. doi: 10.1371/journal.pone.0240996 (PMC7580985; doi:10.1371/journal.pone.0240996)
Supplement: Attachment — Submitted filename: PONE-D-20-18642_reviewer.pdf [file pone.0240996.s007.pdf]

PLOS ONE

Biological invasions alter environmental microbiomes: a meta-analysis

--Manuscript Draft--

**Manuscript Number:**	PONE-D-20-18642
**Article Type:**	Research Article
**Full Title:**	Biological invasions alter environmental microbiomes: a meta-analysis
**Short Title:**	Biological invasions alter microbiomes
**Corresponding Author:**	Antonino Malacrinò<br>Ohio State University<br>Columbus, OH UNITED STATES
**Keywords:**	invasive species; metabarcoding; amplicon-sequencing; 16S
**Abstract:**	Biological invasions impact both agricultural and natural systems. The damage can be quantified in terms of both economic loss and reduction of biodiversity. Although the literature is quite rich about the impact of invasive species on plant and animal communities, their impact on environmental microbiomes is underexplored. Here, we re-analyze publicly available data using a common framework to create a global synthesis of the effects of biological invasions on environmental microbial communities. Our findings suggest that non-native species are responsible for the loss of microbial diversity and shifts in the structure of microbial populations. Therefore, the impact of biological invasions on native ecosystems might be more pervasive than previously thought, influencing both macro- and micro-biomes. We also identified gaps in the literature which encourage research on a wider variety of environments and invaders, and the influence of invaders across seasons and geographical ranges.
**Order of Authors:**	Antonino Malacrinò
	Victoria A. Sadowski
	Tvisha K. Martin
	Nathalia Cavichiolli de Oliveira
	Ian J. Brackett
	James D. Feller
	Kristian J. Harris
	Orlando Combita Heredia
	Rosa Vescio
	Alison E. Bennett
**Additional Information:**	
**Question**	**Response**
**Financial Disclosure**<br><br>Enter a financial disclosure statement that describes the sources of funding for the work included in this submission. Review the submission guidelines for detailed requirements. View published research articles from *PLOS ONE* for specific examples.<br><br>This statement is required for submission and **will appear in the published article** if	The author(s) received no specific funding for this work.

Powered by Editorial Manager® and ProduXion Manager® from Aries Systems Corporation

the submission is accepted. Please make sure it is accurate.<br><br>**Unfunded studies**<br>Enter: *The author(s) received no specific funding for this work.*<br><br>**Funded studies**<br>Enter a statement with the following details:<br>• Initials of the authors who received each award<br>• Grant numbers awarded to each author<br>• The full name of each funder<br>• URL of each funder website<br>• Did the sponsors or funders play any role in the study design, data collection and analysis, decision to publish, or preparation of the manuscript?<br>• **NO** - Include this sentence at the end of your statement: *The funders had no role in study design, data collection and analysis, decision to publish, or preparation of the manuscript.*<br>• **YES** - Specify the role(s) played.<br><br>* typeset	
**Competing Interests**<br><br>Use the instructions below to enter a competing interest statement for this submission. On behalf of all authors, disclose any competing interests that could be perceived to bias this work—acknowledging all financial support and any other relevant financial or non-financial competing interests.<br><br>This statement **will appear in the published article** if the submission is accepted. Please make sure it is accurate. View published research articles from *PLOS ONE* for specific examples.	The authors have declared that no competing interests exist.

Powered by Editorial Manager® and ProduXion Manager® from Aries Systems Corporation

**NO authors have competing interests**<br><br>Enter: *The authors have declared that no competing interests exist.*<br><br>**Authors with competing interests**<br><br>Enter competing interest details beginning with this statement:<br><br>*I have read the journal's policy and the authors of this manuscript have the following competing interests: [insert competing interests here]*<br><br>* typeset	
**Ethics Statement**<br><br>Enter an ethics statement for this submission. This statement is required if the study involved:<br><br>• Human participants<br>• Human specimens or tissue<br>• Vertebrate animals or cephalopods<br>• Vertebrate embryos or tissues<br>• Field research<br><br>Write "N/A" if the submission does not require an ethics statement.<br><br>General guidance is provided below. Consult the submission guidelines for detailed instructions. **Make sure that all information entered here is included in the Methods section of the manuscript.**	N/A

Powered by Editorial Manager® and ProduXion Manager® from Aries Systems Corporation

**Format for specific study types**<br><br>**Human Subject Research (involving human participants and/or tissue)**<br>• Give the name of the institutional review board or ethics committee that approved the study<br>• Include the approval number and/or a statement indicating approval of this research<br>• Indicate the form of consent obtained (written/oral) or the reason that consent was not obtained (e.g. the data were analyzed anonymously)<br><br>**Animal Research (involving vertebrate animals, embryos or tissues)**<br>• Provide the name of the Institutional Animal Care and Use Committee (IACUC) or other relevant ethics board that reviewed the study protocol, and indicate whether they approved this research or granted a formal waiver of ethical approval<br>• Include an approval number if one was obtained<br>• If the study involved *non-human primates*, add *additional details* about animal welfare and steps taken to ameliorate suffering<br>• If anesthesia, euthanasia, or any kind of animal sacrifice is part of the study, include briefly which substances and/or methods were applied<br><br>**Field Research**<br><br>Include the following details if this study involves the collection of plant, animal, or other materials from a natural setting:<br>• Field permit number<br>• Name of the institution or relevant body that granted permission	
**Data Availability**<br><br>Authors are required to make all data underlying the findings described fully available, without restriction, and from the time of publication. PLOS allows rare exceptions to address legal and ethical concerns. See the PLOS Data Policy and FAQ for detailed information.	Yes - all data are fully available without restriction

A Data Availability Statement describing where the data can be found is required at submission. Your answers to this question constitute the Data Availability Statement and **will be published in the article**, if accepted.<br><br>**Important:** Stating 'data available on request from the author' is not sufficient. If your data are only available upon request, select 'No' for the first question and explain your exceptional situation in the text box.<br><br>Do the authors confirm that all data underlying the findings described in their manuscript are fully available without restriction?	
**Describe where the data may be found in full sentences. If you are copying our sample text, replace any instances of XXX with the appropriate details.**<br><br>• If the data are **held or will be held in a public repository**, include URLs, accession numbers or DOIs. If this information will only be available after acceptance, indicate this by ticking the box below. For example: *All XXX files are available from the XXX database (accession number(s) XXX, XXX.).*<br>• If the data are all contained **within the manuscript and/or Supporting Information files**, enter the following: *All relevant data are within the manuscript and its Supporting Information files.*<br>• If neither of these applies but you are able to provide **details of access elsewhere**, with or without limitations, please do so. For example:<br><br>*Data cannot be shared publicly because of [XXX]. Data are available from the XXX Institutional Data Access / Ethics Committee (contact via XXX) for researchers who meet the criteria for access to confidential data.*<br><br>*The data underlying the results presented in the study are available from (include the name of the third party*	Raw data from single studies is publicly available. The code used to perform our analyses has been deposited on GitHub: https://github.com/amalacrino/Biol_Invasion_Microb_MA

and contact information or URL).<br>• This text is appropriate if the data are owned by a third party and authors do not have permission to share the data.<br><br>* typeset	
Additional data availability information:	

Powered by Editorial Manager® and ProduXion Manager® from Aries Systems Corporation

Manuscript Click here to download Manuscript Manuscript.docx

Biological invasions alter environmental microbiomes: a meta-analysis

Antonino Malacrinò[1,*], Victoria A. Sadowski[1], Tvisha K. Martin[2], Nathalia Cavichiolli de Oliveira[3], Ian J. Brackett[1], James D. Feller[1], Kristian J. Harris[1], Orlando Combita Heredia[1], Rosa Vescio[1,4], Alison E. Bennett[1]

[1] Department of Evolution, Ecology and Organismal Biology, The Ohio State University, Columbus (OH)

[2] College of Food, Agricultural and Environmental Sciences, The Ohio State University, Columbus (OH)

[3] Department of Entomology, University of Sao Paulo, Piracicaba (SP), Brazil

[4] Dipartimento AGRARIA, Università degli Studi Mediterranea, Reggio Calabria, Italy

*Corresponding author.

Email: malacrino.1@osu.edu; antonino.malacrino@gmail.com (AM)

Abstract

Biological invasions impact both agricultural and natural systems. The damage can be quantified in terms of both economic loss and reduction of biodiversity. Although the literature is quite rich about the impact of invasive species on plant and animal communities, their impact on environmental microbiomes is underexplored. Here, we re-analyze publicly available data using a common framework to create a global synthesis of the effects of biological invasions on environmental microbial communities. Our findings suggest that non-native species are responsible for the loss of microbial diversity and shifts in the structure of microbial populations. Therefore, the impact of biological invasions on native ecosystems might be more pervasive than previously thought, influencing both macro- and micro-biomes. We also identified gaps in the literature which encourage research on a wider variety of environments and invaders, and the influence of invaders across seasons and geographical ranges.

Introduction

Biological invasions have severe impacts on biodiversity, community composition and ecosystem functions [1–5]. Invasive plants can alter many important ecosystem functions including the nitrogen cycle [6], carbon cycle, and decomposition. For example, invasion by the plant Amur Honeysuckle altered the decomposition rate in the invaded environment likely through changes in litter quality [7]. Exotic snails have been found to alter carbon and nitrogen fluxes in freshwater systems through their consumption/excretion activity [8]. These functions are provided by environmental microbiomes. Yet, despite the implications for ecosystem

functioning, we are still learning the consequences of biological invasions on environmental microbiomes.

Previous studies have shown biological invasions can impact the diversity and taxonomical structure of environmental microbiomes. For example, we often see a shift in soil microbiota following invasion by non-native plant species [9–19]. Removal of feral pigs increased the diversity of soil bacterial communities and shifted their structure [20], and invasive crustaceans [21], mussels [22] and jellyfish [23] produce changes in the structure of water microbiomes. However, shifts in environmental microbiome as consequence of biological invasions do not always occur. For example, invasion by the plants *Robinia pseudoacacia* [24], *Eucalyptus* sp. [25], *Vincetoxicum rossicum* [26] did not alter the structure of soil microbial communities. Also, microcosms exposed to the simultaneous invasion of multiple plant species [27–29] did not alter soil microbiomes. Similarly, soil microbiome structure in microcosms did not change with the addition of the invasive earthworm *Aporrectodea trapezoides* [30]. Several of these studies used techniques (e.g. DGGE, PLFA, t-RFLP) that limit fine scale investigations of biological invasions on environmental microbiome diversity and taxonomical composition. Among the studies using high-throughput amplicon-sequencing techniques, most did not find changes in microbiome diversity [13,15,18,19,21,25,27,28,30], few reported a decrease of microbial diversity in response to invasion [16,20,29], and fewer still reported an increase [11,14]. Thus, there is still little consensus on the effects of biological invasions on the diversity and taxonomical structure of the environmental microbiomes, both tied to the stability and function of microbial communities [31,32].

Our ability to draw broad conclusions from published studies is limited, because individual studies have occurred within a limited geographical range or with a limited group of species. Meta-analyses of published biological means have long enabled more robust conclusions than individual studies [33–36]. However, the meta-analytic approach has less frequently been applied to amplicon-sequencing data that represent environmental microbiome community composition. The majority of meta-analytic metabarcoding studies have occurred in the medical sciences [37–45]. This approach can be successfully used to address ecological questions. For example, meta-analytic metabarcoding studies have found common patterns in the structure of indoor microbiomes [46] and freshwater eukaryotes [47]. Shade et al. [48] also used a meta-analysis of metabarcoding datasets from different environments highlighting a time-dependent structure of microbiomes. A meta-analytic approach has also been used to test the effects of stressors (e.g. water availability, temperature, heavy metals) on environmental microbiomes [49]. Thus meta-analyses on microbiome data have a striking potential to address global-scale questions, generate new hypotheses and model common patterns [50], because they provide across study comparisons [39,51,52].

Here, we aim to test whether the effect of biological invasion on environmental microbiomes can be generalized or is idiosyncratic. To do so, we collected publicly available data and re-analyzed this data under a common framework. We tested the effect of invasive species on the diversity and structure of environmental microbiomes with the hypothesis that the presence of invasive species will decrease microbial diversity, and alter the composition of the environmental microbiome. We then investigated whether certain taxonomical groups are more responsive to biological invasions.

Methods

Data collection

We searched for metabarcoding studies that evaluated the effect of biological invasions on environmental microbiomes, and compared invaded and non-invaded habitats. Our literature search for this study was conducted using Web of Science Core Collection (accessed on March 6th, 2020) using the keywords "Invasive speci*" and "microbio*" published between 2010-2020, and found 1,471 studies. Two additional studies were added by searching the same keywords on Google Scholar (Fig S1). Records were manually filtered based on the study design appropriate for our research question. This step yielded 22 studies, and we further filtered these studies based on data availability in public repositories. When data was not available, we attempted to contact the corresponding author. Finally we selected only studies that used the 16S rRNA marker gene, primer pair 515F/806R [53] or 341F/785R [54], and Illumina MiSeq sequencing platform. After discarding studies that failed quality checks (see below), we were able to include a total of five studies (Tab. 1), summing up to a total of 356 samples. Three studies focused on invasive plants, and the remaining studies focused on a mammal and a mussel (Tab. 1). The invasive mussel study [22] was the only study performed in an aquatic environment, while the remaining studies examined soil environments.

Table 1. Summary of studies included in the meta-analysis

Study ID	Invasive organism	Species	Invaded environment	Reference
MPG13011	Plant	*Agropyron cristatum*, *Bromus tectorum*, *Sisymbrium altissimum*, *Erodium cicutarium* and *Poa bulbosa*	Soil	Gibbons et al. [28]
MPG87547	Mammal	*Sus scrofa*	Soil	Wehr et al. [20]
PRJNA296487	Plant	*Microstegium vimineum*, *Rhamnus davurica* and *Ailanthus altissima*	Soil	Rodrigues et al. [19]
PRJNA320310	Plant	*Artemisia rothrockii*	Soil	Collins et al. [11]
PRJNA385848	Mussel	*Dreissena bugensis*	Water	Denef et al. [22]

We took the following steps to alleviate some of the potential sources of bias due to studies performed in different labs, using different protocols and sequenced on different instruments. First, all studies included were performed using the Illumina MiSeq platform, in order to reduce the potential bias that might be generated by directly comparing data obtained from different platforms. Second, all studies targeted the same region of the 16S rRNA, as several

primer pairs targeting different regions are currently published and widely used. Three out of five papers we considered in our analysis used the 515F/806R primer pair [53], while two used the 341F/785R [54]. Although there might be small differences between them, they overlap in the V4 region of 16S gene (Fig. S2), so we feel confident that the chance of including spurious OTUs in our analysis is quite negligible. To account for study-specific variances due to small differences in sampling procedures and lab protocols, we also included the study itself, the environment where the study was performed (i.e., soil or water) and the identity of the invasive species as stratification variables in the PERMANOVA and as random factors in our linear model. This allowed us to ensure that our results are not biased by study-specific features.

Once the papers were selected, we assigned each a “Study ID” and collected meta-data from each sample in each study (invasive species, type of organism, invaded environment). We then downloaded data from repositories using SRA Toolkit 2.10.4 for data on the SRA databases, or by directly downloading files from the MG-RAST database.

Data processing and analysis

Paired-end reads were merged using FLASH 1.2.11 [55] and data were processed using QIIME 1.9.1 [56]. Quality-filtering of reads was performed using default parameters, binning OTUs and discarding chimeric sequences identified with VSEARCH 2.14.2 [57]. Taxonomy for representative sequences was determined by querying against the SILVA database v132 [58] using the BLAST method. A phylogeny was obtained by aligning representative sequences using MAFFT v7.464 [59] and reconstructing a phylogenetic tree using FastTree [60].

Data analysis was performed using R statistical software 3.5 [61] with the packages *phyloseq* [62] and *vegan* [63]. Read counts were normalized using DESeq2 v1.22.2 [64] prior to data analysis. Singletons and sequences classified as chloroplast were excluded, as well as samples which had less than 5000 sequence counts. Shannon diversity was fit to a linear mixed-effects model specifying *sample type* (invaded or control), *organism* (plant, mammal, mussel), and their interactions as fixed factors with *studyID* and *environment* (soil or water) included as random factors. Models were fit using the *lmer*() function under the *lme4* package [65] and the package *emmeans* was used to infer pairwise contrasts (corrected using False Discovery Rate, FDR). Furthermore, we explored the effects of *sample type* and *organism* on the structure of the microbial communities using a multivariate approach. Distances between pairs of samples, in terms of community composition, were calculated using a Unifrac matrix, and then visualized using an RDA procedure. Differences between sample groups were inferred through PERMANOVA multivariate analysis (999 permutations stratified at level of *studyID*, *environment* and *identity of invasive species*). Pairwise contrasts from PERMANOVA were subjected to FDR correction. Finally, the relative abundance of each bacterial family was fit using the *lmer*() function to test the effects of *sample type* (invaded or control) on single taxa, with *studyID*, *organism* (plant, mammal, mussel) and *environment* included as random factors.

Results

Our search yielded 5 studies with an appropriate experimental design and available data, for a total of 356 samples. A few samples failed quality checks and we further considered 335 samples for downstream analyses. Sequences clustered into 22831 OTUs, after quality checks,

removal of singletons and “chloroplast” reads, with an average of 61776.92 reads per sample. Although the number of OTUs might seem high, it is important to consider that we are analyzing samples across multiple environments (soil and water) and from different geographical regions which we would expect to increase richness.

Biological invasions led to a reduction in Shannon diversity (χ^2= 3.85, df=1, *P*=0.04, Fig 1A). We also found biological invasions altered microbiome community composition in the invaded environment compared to the control (Tab. 2, Fig 1B). The type of invasive organism (plant, mammal, or mussel) produced a different community structure (pairwise *P*<0.01, FDR corrected). A deeper analysis of bacterial families (Fig. 1C, Table S2) revealed that some taxonomic groups are significantly more abundant in invaded environments (Blastocatellaceae, Chitinophagaceae, Nitrosomonadaceae, Pirellulaceae, Sphingomonadaceae), while others are more abundant in non-invaded samples (Acetobacteraceae, Beijerinckiaceae, Gemmataceae, Micromonosporaceae, Pedosphaeraceae, Solibacteraceae, Solirubrobacteraceae).

Table 2. Results from PERMANOVA analysis testing the effects of *sample type* (invaded/control), *organism group* (plant, mammal, mussel) and their interaction on microbial community composition. The factors *studyID* (unique for each study) and *environment* (soil or water) were used as strata to constrain permutations.

Factor	df	R^2	F	P
Sample type (Invaded/Control)	1	0.011	6.68	<0.001
Organism group (plant, mammal, mussel)	3	0.411	118.86	<0.001
Sample type * Organism group	3	0.007	2.1	0.02

Fig 1. (A) Comparison of Shannon index between control and invaded environments. (B) RDA ordination using a Bray-Curtis distance matrix of samples. (C) Comparison of the relative abundance of microbial classes between control and invaded environments. * $P<0.05$, ** $P<0.01$, *** $P<0.001$

Discussion

We show biological invasions decrease the diversity of environmental microbiomes. Thus, understanding the impact of biological invasions on environmental microbiomes is of high priority if we want to preserve ecosystem functions [66]. While several studies have investigated the effects of species invasions on environmental microbiomes, we still lack a generalized consensus across different environmental microbiomes and systems. Previous studies have found that invasive species increased environmental microbial diversity [11,14], while others

reported a decrease [16,20,29]. But the majority of studies did not analyze the microbial diversity, because they used techniques that did not allow such analysis, or reported no changes [9,10,12,13,15,17–19,21–28,30]. Within the studies included in our analysis, invasion by feral pigs decreased soil microbial diversity, while invasion by *Artemisia rothrockii* increased soil microbial diversity. The remaining three studies in our analysis reported no effects of biological invasions on environmental microbiome diversity. Microbial diversity is tied to the function of microbiomes, and changes in diversity can reflect changes in function [67–69]. Changes in microbial diversity and function do not always have the same direction [70] and this might explain the discrepancy between our results and other studies.

In contrast to diversity, our report of changes in community composition was relatively consistent with the published literature and the individual results of the studies we analyzed. Most studies of the influence of biological invasions on environmental microbiomes found that biological invasions alter environmental microbial community composition. However, some previous reports did not report changes [24–30], including the study by Gibbons et al. [28] considered in our analysis. This variation may be due to individual effects of organisms on the environment. For example, invasive plants may alter soil microbiome composition through root exudates [5], and invasive mussels may alter water microbiome composition via bacterial removal through their feeding activity [22]. Thus, reported influences on community composition are more consistent. Alternatively changes in community composition might due to the response of some bacterial groups to environmental disturbance. The bacterial families that we found to be differentially abundant between the invaded and control environments have diverse ecological functions ranging from nitrogen fixation and carbohydrate metabolism to

antimicrobial properties. Many of the families that showed a significant difference between invaded and control environments have species members that play important roles at various points during nitrogen and carbon cycling (i.e. Nitrosomonadaceae, Acetobacteraceae, Chitinophagaceae, Micromonosporaceae, Gemmataceae, Beijerinckiaceae, Pirellulaceae) [71–81]. However, nitrogen fixing and carbohydrate degrading bacteria did not have a unified response to invaded environments as some increased and others decreased in abundance in invaded environments. Many nitrogen fixing bacteria have been shown to respond to environmental disturbance, such as Acidobacteria abundances during forest to pasture conversions or Pirellulaceae's response to the presence of microplastics [82,83]. Thus, changes in environmental microbiome community composition appear to be linked to changes in ecosystem functions, although this pattern is not yet predictable for all functions and taxa.

Few previous studies on biological invasions have reported details on the differential abundance of taxa, and among these we found limited general consensus. For example, some studies report a decrease in abundance of bacteria associated with nitrogen cycling (e.g. Nitrosphaeria, Nitrospira, Nitrosomonadales) [13,14,19], while others report an increase of Nitrosomonadaceae following invasion [30]. In our study some groups associated with the nitrogen cycle were positively associated with biological invasions (i.e. Nitrosomonadaceae, Pirellulaceae, Chitinophagaceae) while others were negatively associated (Beijerinckiaceae, Micromonosporaceae). Unfortunately, amplicon-based sequencing has a limited power to infer changes in the functions of microbiomes. Future metagenomic and metatranscriptomic studies are needed to investigate whether biological invasions alter gene content or gene expression of environmental microbiomes, and whether this reflects changes in biogeochemical cycling.

Meta-analyses are also useful to highlight gaps in the literature, and here we highlight some aspects that warrant further investigation. We identified a large gap in the availability of sequencing data from multiple types of environments and types of invasive species. For our analysis almost all available data came from two environments: four sets of data came from soil and one came from freshwater. Greater effort is needed for sample collection from invasions in both freshwater and marine environments. Without sufficient diversity of sample environments, it is impossible to tell whether microbial shifts following an invasion are unique to an invaded environment. Second, in our analysis the majority of data came from one type of invasive species: plants. Noticeably absent from our dataset were invasions by insects, fish, and amphibians. Sequencing data is needed from a larger number of invasive species to allow us to broadly assess shifts in microbial community structure. A third gap we identified was the lack of spatial and temporal resolution. Almost all of the initially identified 22 studies we assessed were also restricted to one season of sampling and were conducted in the Northern Hemisphere. Thus, it is impossible to validate the influence of latitude with existing datasets or explore how seasonality and biological invasions interact to modulate microbial communities. Thus, there are a number of opportunities for future research on how biological invasions alter environmental microbial communities.

Here we analyzed 16S amplicon sequencing data from five studies and show that biological invasions influence both the diversity and the structure of environmental microbiomes. We identified a number of gaps in our knowledge, including the need to assess a wider range of environments, invasive species, temporal variation, and latitudinal variation. We also

demonstrate the power of re-analysis of publicly available datasets using a common pipeline which benefited from open-data initiatives.

Acknowldgements

We would like to thank Kali Mattingly (Ohio State University, USA) and Davide Rassati (Università degli Studi di Padova, Italy) for their helpful comments on the manuscript.

Data accessibility

Raw data from single studies is publicly available. The code used to perform our analyses has been deposited on GitHub: https://github.com/amalacrino/ Biol_Invasion_Microb_MA

References

1. Chapin III FS, Zavaleta ES, Eviner VT, Naylor RL, Vitousek PM, Reynolds HL, et al. Consequences of changing biodiversity. Nature. 2000;405: 234–242. doi:10.1038/35012241
2. Pejchar L, Mooney HA. Invasive species, ecosystem services and human well-being. Trends Ecol Evol. 2009;24: 497–504. doi:10.1016/j.tree.2009.03.016
3. Shackleton RT, Shackleton CM, Kull CA. The role of invasive alien species in shaping local livelihoods and human well-being: A review. J Environ Manage. 2019;229: 145–157. doi:10.1016/j.jenvman.2018.05.007
4. Charles H, Dukes JS. Impacts of Invasive Species on Ecosystem Services. Biological Invasions. Berlin, Heidelberg: Springer Berlin Heidelberg; 2008. pp. 217–237. doi:10.1007/978-3-540-36920-2_13
5. Weidenhamer JD, Callaway RM. Direct and Indirect Effects of Invasive Plants on Soil Chemistry and Ecosystem Function. J Chem Ecol. 2010;36: 59–69. doi:10.1007/s10886-009-9735-0
6. Lee MR, Bernhardt ES, van Bodegom PM, Cornelissen JHC, Kattge J, Laughlin DC, et al. Invasive species' leaf traits and dissimilarity from natives shape their impact on nitrogen cycling: a meta-analysis. New Phytol. 2017;213: 128–139. doi:10.1111/nph.14115
7. Arthur MA, Bray SR, Kuchle CR, McEwan RW. The influence of the invasive shrub, Lonicera maackii, on leaf decomposition and microbial community dynamics. Plant Ecol. 2012;213: 1571–1582. doi:10.1007/s11258-012-0112-7
8. Hall Jr RO, Tank JL, Dybdahl MF. Exotic snails dominate nitrogen and carbon cycling in a

highly productive stream. Front Ecol Environ. 2003;1: 407–411. doi:10.1890/1540-9295(2003)001[0407:ESDNAC]2.0.CO;2

9. Lazzaro L, Giuliani C, Fabiani A, Agnelli AE, Pastorelli R, Lagomarsino A, et al. Soil and plant changing after invasion: The case of Acacia dealbata in a Mediterranean ecosystem. Sci Total Environ. 2014;497–498: 491–498. doi:10.1016/j.scitotenv.2014.08.014

10. Malinich E, Lynn-Bell N, Kourtev PS. The effect of the invasive Elaeagnus umbellata on soil microbial communities depends on proximity of soils to plants. Ecosphere. 2017;8: e01827. doi:10.1002/ecs2.1827

11. Collins CG, Carey CJ, Aronson EL, Kopp CW, Diez JM. Direct and indirect effects of native range expansion on soil microbial community structure and function. Austin A, editor. J Ecol. 2016;104: 1271–1283. doi:10.1111/1365-2745.12616

12. Lorenzo P, Pereira CS, Rodríguez-Echeverría S. Differential impact on soil microbes of allelopathic compounds released by the invasive Acacia dealbata Link. Soil Biol Biochem. 2013;57: 156–163. doi:10.1016/j.soilbio.2012.08.018

13. Parsons LS, Sayre J, Ender C, Rodrigues JLM, Barberán A. Soil microbial communities in restored and unrestored coastal dune ecosystems in California. Restor Ecol. 2020; rec.13101. doi:10.1111/rec.13101

14. Piper CL, Siciliano SD, Winsley T, Lamb EG. Smooth brome invasion increases rare soil bacterial species prevalence, bacterial species richness and evenness. De Deyn G, editor. J Ecol. 2015;103: 386–396. doi:10.1111/1365-2745.12356

15. Zhang H-Y, Goncalves P, Copeland E, Qi S-S, Dai Z-C, Li G-L, et al. Invasion by the weed Conyza canadensis alters soil nutrient supply and shifts microbiota structure. Soil Biol

Biochem. 2020;143: 107739. doi:10.1016/j.soilbio.2020.107739

16. Le Roux JJ, Ellis AG, van Zyl L-M, Hosking ND, Keet J-H, Yannelli FA. Importance of soil legacy effects and successful mutualistic interactions during Australian acacia invasions in nutrient-poor environments. Aerts R, editor. J Ecol. 2018;106: 2071–2081. doi:10.1111/1365-2745.12965

17. Brunel C, Beifen Y, Pouteau R, Li J, van Kleunen M. Responses of Rhizospheric Microbial Communities of Native and Alien Plant Species to Cuscuta Parasitism. Microb Ecol. 2020;79: 617–630. doi:10.1007/s00248-019-01438-z

18. Pickett B, Irvine IC, Bullock E, Arogyaswamy K, Aronson E. Legacy effects of invasive grass impact soil microbes and native shrub growth. Invasive Plant Sci Manag. 2019;12: 22–35. doi:10.1017/inp.2018.32

19. Rodrigues RR, Pineda RP, Barney JN, Nilsen ET, Barrett JE, Williams MA. Plant Invasions Associated with Change in Root-Zone Microbial Community Structure and Diversity. Liu J, editor. PLoS One. 2015;10: e0141424. doi:10.1371/journal.pone.0141424

20. Wehr NH, Kinney KM, Nguyen NH, Giardina CP, Litton CM. Changes in soil bacterial community diversity following the removal of invasive feral pigs from a Hawaiian tropical montane wet forest. Sci Rep. 2019;9: 14681. doi:10.1038/s41598-019-48922-7

21. Boeker C, Geist J. Effects of invasive and indigenous amphipods on physico-chemical and microbial properties in freshwater substrates. Aquat Ecol. 2015;49: 467–480. doi:10.1007/s10452-015-9539-y

22. Denef VJ, Carrick HJ, Cavaletto J, Chiang E, Johengen TH, Vanderploeg HA. Lake Bacterial Assemblage Composition Is Sensitive to Biological Disturbance Caused by an Invasive Filter

Feeder. Kent AD, editor. mSphere. 2017;2. doi:10.1128/mSphere.00189-17

23. Manzari C, Fosso B, Marzano M, Annese A, Caprioli R, D'Erchia AM, et al. The influence of invasive jellyfish blooms on the aquatic microbiome in a coastal lagoon (Varano, SE Italy) detected by an Illumina-based deep sequencing strategy. Biol Invasions. 2015;17: 923–940. doi:10.1007/s10530-014-0810-2

24. Lazzaro L, Mazza G, D'Errico G, Fabiani A, Giuliani C, Inghilesi AF, et al. How ecosystems change following invasion by Robinia pseudoacacia: Insights from soil chemical properties and soil microbial, nematode, microarthropod and plant communities. Sci Total Environ. 2018;622–623: 1509–1518. doi:10.1016/j.scitotenv.2017.10.017

25. Hernández-Gómez O, Byrne AQ, Gunderson AR, Jenkinson TS, Noss CF, Rothstein AP, et al. Invasive vegetation affects amphibian skin microbiota and body condition. PeerJ. 2020;8: e8549. doi:10.7717/peerj.8549

26. Thompson GL, Bell TH, Kao-Kniffin J. Rethinking Invasion Impacts across Multiple Field Sites Using European Swallowwort (Vincetoxicum rossicum) as a Model Invader. Invasive Plant Sci Manag. 2018;11: 109–116. doi:10.1017/inp.2018.22

27. Carey CJ, Beman JM, Eviner VT, Malmstrom CM, Hart SC. Soil microbial community structure is unaltered by plant invasion, vegetation clipping, and nitrogen fertilization in experimental semi-arid grasslands. Front Microbiol. 2015;6. doi:10.3389/fmicb.2015.00466

28. Gibbons SM, Lekberg Y, Mummey DL, Sangwan N, Ramsey PW, Gilbert JA. Invasive Plants Rapidly Reshape Soil Properties in a Grassland Ecosystem. Shade A, editor. mSystems. 2017;2. doi:10.1128/mSystems.00178-16

29. Gomes SIF, Merckx VSFT, Hynson NA. Biological invasions increase the richness of arbuscular mycorrhizal fungi from a Hawaiian subtropical ecosystem. Biol Invasions. 2018;20: 2421–2437. doi:10.1007/s10530-018-1710-7

30. de Menezes AB, Prendergast-Miller MT, Macdonald LM, Toscas P, Baker G, Farrell M, et al. Earthworm-induced shifts in microbial diversity in soils with rare versus established invasive earthworm populations. FEMS Microbiol Ecol. 2018;94. doi:10.1093/femsec/fiy051

31. Bier RL, Bernhardt ES, Boot CM, Graham EB, Hall EK, Lennon JT, et al. Linking microbial community structure and microbial processes: an empirical and conceptual overview. Muyzer G, editor. FEMS Microbiol Ecol. 2015;91: fiv113. doi:10.1093/femsec/fiv113

32. Gibbons SM, Gilbert JA. Microbial diversity — exploration of natural ecosystems and microbiomes. Curr Opin Genet Dev. 2015;35: 66–72. doi:10.1016/j.gde.2015.10.003

33. Gurevitch J, Koricheva J, Nakagawa S, Stewart G. Meta-analysis and the science of research synthesis. Nature. 2018;555: 175–182. doi:10.1038/nature25753

34. Nakagawa S, Poulin R. Meta-analytic insights into evolutionary ecology: an introduction and synthesis. Evol Ecol. 2012;26: 1085–1099. doi:10.1007/s10682-012-9593-z

35. Nakagawa S, Santos ESA. Methodological issues and advances in biological meta-analysis. Evol Ecol. 2012;26: 1253–1274. doi:10.1007/s10682-012-9555-5

36. Senior AM, Grueber CE, Kamiya T, Lagisz M, O’Dwyer K, Santos ESA, et al. Heterogeneity in ecological and evolutionary meta-analyses: its magnitude and implications. Ecology. 2016;97: 3293–3299. doi:10.1002/ecy.1591

37. Lozupone CA, Stombaugh J, Gonzalez A, Ackermann G, Wendel D, Vazquez-Baeza Y, et al.

Meta-analyses of studies of the human microbiota. Genome Res. 2013;23: 1704–1714. doi:10.1101/gr.151803.112

38. Walters WA, Xu Z, Knight R. Meta-analyses of human gut microbes associated with obesity and IBD. FEBS Lett. 2014;588: 4223–4233. doi:10.1016/j.febslet.2014.09.039

39. Duvallet C, Gibbons SM, Gurry T, Irizarry RA, Alm EJ. Meta-analysis of gut microbiome studies identifies disease-specific and shared responses. Nat Commun. 2017;8: 1784. doi:10.1038/s41467-017-01973-8

40. Koren O, Knights D, Gonzalez A, Waldron L, Segata N, Knight R, et al. A Guide to Enterotypes across the Human Body: Meta-Analysis of Microbial Community Structures in Human Microbiome Datasets. Eisen JA, editor. PLoS Comput Biol. 2013;9: e1002863. doi:10.1371/journal.pcbi.1002863

41. Krych L, Hansen CHF, Hansen AK, van den Berg FWJ, Nielsen DS. Quantitatively Different, yet Qualitatively Alike: A Meta-Analysis of the Mouse Core Gut Microbiome with a View towards the Human Gut Microbiome. Bereswill S, editor. PLoS One. 2013;8: e62578. doi:10.1371/journal.pone.0062578

42. Wang J, Kurilshikov A, Radjabzadeh D, Turpin W, Croitoru K, Bonder MJ, et al. Meta-analysis of human genome-microbiome association studies: the MiBioGen consortium initiative. Microbiome. 2018;6: 101. doi:10.1186/s40168-018-0479-3

43. Jiao N, Baker SS, Nugent CA, Tsompana M, Cai L, Wang Y, et al. Gut microbiome may contribute to insulin resistance and systemic inflammation in obese rodents: a meta-analysis. Physiol Genomics. 2018;50: 244–254. doi:10.1152/physiolgenomics.00114.2017

44. Wirbel J, Pyl PT, Kartal E, Zych K, Kashani A, Milanese A, et al. Meta-analysis of fecal

metagenomes reveals global microbial signatures that are specific for colorectal cancer. Nat Med. 2019;25: 679–689. doi:10.1038/s41591-019-0406-6

45. Bisanz JE, Upadhyay V, Turnbaugh JA, Ly K, Turnbaugh PJ. Meta-Analysis Reveals Reproducible Gut Microbiome Alterations in Response to a High-Fat Diet. Cell Host Microbe. 2019;26: 265-272.e4. doi:10.1016/j.chom.2019.06.013

46. Adams RI, Bateman AC, Bik HM, Meadow JF. Microbiota of the indoor environment: a meta-analysis. Microbiome. 2015;3: 49. doi:10.1186/s40168-015-0108-3

47. Debroas D, Domaizon I, Humbert J-F, Jardillier L, Lepère C, Oudart A, et al. Overview of freshwater microbial eukaryotes diversity: a first analysis of publicly available metabarcoding data. FEMS Microbiol Ecol. 2017;93. doi:10.1093/femsec/fix023

48. Shade A, Gregory Caporaso J, Handelsman J, Knight R, Fierer N. A meta-analysis of changes in bacterial and archaeal communities with time. ISME J. 2013;7: 1493–1506. doi:10.1038/ismej.2013.54

49. Rocca JD, Simonin M, Blaszczak JR, Ernakovich JG, Gibbons SM, Midani FS, et al. The Microbiome Stress Project: Toward a Global Meta-Analysis of Environmental Stressors and Their Effects on Microbial Communities. Front Microbiol. 2019;9. doi:10.3389/fmicb.2018.03272

50. Duvallet C. Meta-analysis generates and prioritizes hypotheses for translational microbiome research. Microb Biotechnol. 2018;11: 273–276. doi:10.1111/1751-7915.13047

51. Drewes JL, White JR, Dejea CM, Fathi P, Iyadorai T, Vadivelu J, et al. High-resolution bacterial 16S rRNA gene profile meta-analysis and biofilm status reveal common colorectal

cancer consortia. npj Biofilms Microbiomes. 2017;3: 34. doi:10.1038/s41522-017-0040-3

52. Ramirez KS, Knight CG, de Hollander M, Brearley FQ, Constantinides B, Cotton A, et al. Detecting macroecological patterns in bacterial communities across independent studies of global soils. Nat Microbiol. 2018;3: 189–196. doi:10.1038/s41564-017-0062-x

53. Caporaso JG, Lauber CL, Walters WA, Berg-Lyons D, Lozupone CA, Turnbaugh PJ, et al. Global patterns of 16S rRNA diversity at a depth of millions of sequences per sample. Proc Natl Acad Sci. 2011;108: 4516–4522. doi:10.1073/pnas.1000080107

54. Klindworth A, Pruesse E, Schweer T, Peplies J, Quast C, Horn M, et al. Evaluation of general 16S ribosomal RNA gene PCR primers for classical and next-generation sequencing-based diversity studies. Nucleic Acids Res. 2013;41: e1–e1. doi:10.1093/nar/gks808

55. Magoc T, Salzberg SL. FLASH: fast length adjustment of short reads to improve genome assemblies. Bioinformatics. 2011;27: 2957–2963. doi:10.1093/bioinformatics/btr507

56. Caporaso JG, Lauber CL, Walters WA, Berg-Lyons D, Huntley J, Fierer N, et al. Ultra-high-throughput microbial community analysis on the Illumina HiSeq and MiSeq platforms. ISME J. 2012;6: 1621–1624. doi:10.1038/ismej.2012.8

57. Rognes T, Flouri T, Nichols B, Quince C, Mahé F. VSEARCH: a versatile open source tool for metagenomics. PeerJ. 2016;4: e2584. doi:10.7717/peerj.2584

58. Quast C, Pruesse E, Yilmaz P, Gerken J, Schweer T, Yarza P, et al. The SILVA ribosomal RNA gene database project: improved data processing and web-based tools. Nucleic Acids Res. 2012;41: D590–D596. doi:10.1093/nar/gks1219

59. Katoh K, Standley DM. MAFFT Multiple Sequence Alignment Software Version 7: Improvements in Performance and Usability. Mol Biol Evol. 2013;30: 772–780.

doi:10.1093/molbev/mst010

60. Price MN, Dehal PS, Arkin AP. FastTree: Computing Large Minimum Evolution Trees with Profiles instead of a Distance Matrix. Mol Biol Evol. 2009;26: 1641–1650. doi:10.1093/molbev/msp077

61. R Core Team. R: A language and environment for statistical computing. Vienna, Austria: R Foundation for Statistical Computing; 2016. 2017.

62. McMurdie PJ, Holmes S. phyloseq: An R package for reproducible interactive analysis and graphics of microbiome census data. Watson M, editor. PLoS One. 2013;8: e61217. doi:10.1371/journal.pone.0061217

63. Dixon P. VEGAN, a package of R functions for community ecology. J Veg Sci. 2003;14: 927–930. doi:10.1111/j.1654-1103.2003.tb02228.x

64. Love MI, Huber W, Anders S. Moderated estimation of fold change and dispersion for RNA-seq data with DESeq2. Genome Biol. 2014;15: 550. doi:10.1186/s13059-014-0550-8

65. Bates D, Mächler M, Bolker B, Walker S. Fitting linear mixed-effects models using lme4. J Stat Softw. 2015;67: 1–48. doi:10.18637/jss.v067.i01

66. Doblas-Miranda E, Martínez-Vilalta J, Lloret F, Álvarez A, Ávila A, Bonet FJ, et al. Reassessing global change research priorities in mediterranean terrestrial ecosystems: how far have we come and where do we go from here? Glob Ecol Biogeogr. 2015;24: 25–43. doi:10.1111/geb.12224

67. Delgado-Baquerizo M, Maestre FT, Reich PB, Jeffries TC, Gaitan JJ, Encinar D, et al. Microbial diversity drives multifunctionality in terrestrial ecosystems. Nat Commun. 2016;7: 10541. doi:10.1038/ncomms10541

68. Miki T, Yokokawa T, Matsui K. Biodiversity and multifunctionality in a microbial community: a novel theoretical approach to quantify functional redundancy. Proc R Soc B Biol Sci. 2014;281: 20132498. doi:10.1098/rspb.2013.2498

69. Philippot L, Spor A, Hénault C, Bru D, Bizouard F, Jones CM, et al. Loss in microbial diversity affects nitrogen cycling in soil. ISME J. 2013;7: 1609–1619. doi:10.1038/ismej.2013.34

70. Mendes LW, Tsai SM, Navarrete AA, de Hollander M, van Veen JA, Kuramae EE. Soil-Borne Microbiome: Linking Diversity to Function. Microb Ecol. 2015;70: 255–265. doi:10.1007/s00248-014-0559-2

71. Chen J, Mo L, Zhang Z, Nan J, Xu D, Chao L, et al. Evaluation of the ecological restoration of a coal mine dump by exploring the characteristics of microbial communities. Appl Soil Ecol. 2020;147: 103430. doi:10.1016/j.apsoil.2019.103430

72. Cheng J, Chen Y, He T, Liao R, Liu R, Yi M, et al. Soil nitrogen leaching decreases as biogas slurry DOC/N ratio increases. Appl Soil Ecol. 2017;111: 105–113. doi:10.1016/j.apsoil.2016.12.001

73. Warneke C, Karl T, Judmaier H, Hansel A, Jordan A, Lindinger W, et al. Acetone, methanol, and other partially oxidized volatile organic emissions from dead plant matter by abiological processes: Significance for atmospheric HO x chemistry. Global Biogeochem Cycles. 1999;13: 9–17. doi:10.1029/98GB02428

74. Kämpfer P, Lodders N, Falsen E. Hydrotalea flava gen. nov., sp. nov., a new member of the phylum Bacteroidetes and allocation of the genera Chitinophaga, Sediminibacterium, Lacibacter, Flavihumibacter, Flavisolibacter, Niabella, Niastella, Segetibacter, Parasegetibacter, Terrimonas, Fer. Int J Syst Evol Microbiol. 2011;61: 518–523.

doi:10.1099/ijs.0.023002-0

75. Madhaiyan M, Poonguzhali S, Senthilkumar M, Pragatheswari D, Lee J-S, Lee K-C. Arachidicoccus rhizosphaerae gen. nov., sp. nov., a plant-growth-promoting bacterium in the family Chitinophagaceae isolated from rhizosphere soil. Int J Syst Evol Microbiol. 2015;65: 578–586. doi:10.1099/ijs.0.069377-0

76. Reis VM, Teixeira KR dos S. Nitrogen fixing bacteria in the family Acetobacteraceae and their role in agriculture. J Basic Microbiol. 2015;55: 931–949. doi:10.1002/jobm.201400898

77. Trujillo ME, Bacigalupe R, Pujic P, Igarashi Y, Benito P, Riesco R, et al. Genome Features of the Endophytic Actinobacterium Micromonospora lupini Strain Lupac 08: On the Process of Adaptation to an Endophytic Life Style? Brüggemann H, editor. PLoS One. 2014;9: e108522. doi:10.1371/journal.pone.0108522

78. Carro L, Nouioui I, Sangal V, Meier-Kolthoff JP, Trujillo ME, Montero-Calasanz M del C, et al. Genome-based classification of micromonosporae with a focus on their biotechnological and ecological potential. Sci Rep. 2018;8: 525. doi:10.1038/s41598-017-17392-0

79. Dedysh SN, Ivanova AA. Planctomycetes in boreal and subarctic wetlands: diversity patterns and potential ecological functions. FEMS Microbiol Ecol. 2019;95. doi:10.1093/femsec/fiy227

80. Wegner C-E, Gorniak L, Riedel S, Westermann M, Küsel K. Lanthanide-Dependent Methylotrophs of the Family Beijerinckiaceae : Physiological and Genomic Insights. Stams AJM, editor. Appl Environ Microbiol. 2019;86. doi:10.1128/AEM.01830-19

81. Neufeld JD, Schäfer H, Cox MJ, Boden R, McDonald IR, Murrell JC. Stable-isotope probing implicates Methylophaga spp and novel Gammaproteobacteria in marine methanol and methylamine metabolism. ISME J. 2007;1: 480–491. doi:10.1038/ismej.2007.65

82. Miao L, Wang P, Hou J, Yao Y, Liu Z, Liu S, et al. Distinct community structure and microbial functions of biofilms colonizing microplastics. Sci Total Environ. 2019;650: 2395–2402. doi:10.1016/j.scitotenv.2018.09.378

83. Navarrete AA, Venturini AM, Meyer KM, Klein AM, Tiedje JM, Bohannan BJM, et al. Differential Response of Acidobacteria Subgroups to Forest-to-Pasture Conversion and Their Biogeographic Patterns in the Western Brazilian Amazon. Front Microbiol. 2015;6. doi:10.3389/fmicb.2015.01443

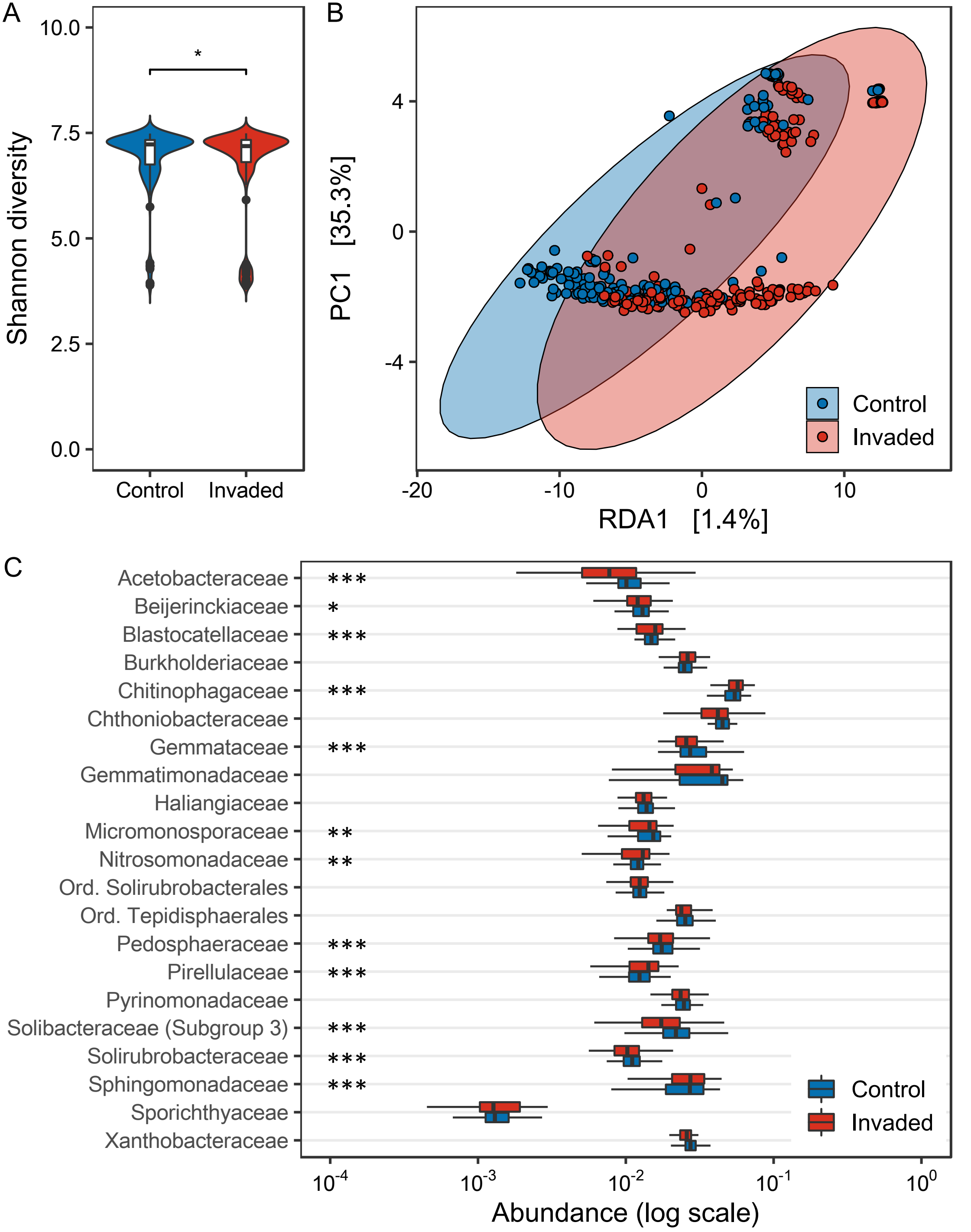

A
10.0
7.5
5.0
2.5
0.0
Shannon diversity
*
Control
Invaded
B
PC1 [35.3%]
4
0
-4
-20
-10
0
10
RDA1 [1.4%]
Control
Invaded
C
Acetobacteraceae ***
Beijerinckiaceae *
Blastocatellaceae ***
Burkholderiaceae
Chitinophagaceae ***
Chthoniobacteraceae
Gemmataceae ***
Gemmatimonadaceae
Haliangiaceae
Micromonosporaceae **
Nitrosomonadaceae **
Ord. Solirubrobacterales
Ord. Tepidisphaerales
Pedosphaeraceae ***
Pirellulaceae ***
Pyrinomonadaceae
Solibacteraceae (Subgroup 3) ***
Solirubrobacteraceae ***
Sphingomonadaceae ***
Sporichthyaceae
Xanthobacteraceae
10^{-4}
10^{-3}
10^{-2}
10^{-1}
10^{0}
Abundance (log scale)
Control
Invaded

Click here to access/download
Supporting Information
Supplementary_material.pdf

Click here to access/download
Preprint PDF
BIORXIV-2020-157883v1-Malacrino.pdf
