# Supplementary material for: Biological invasions alter environmental microbiomes: A meta-analysis
Source: PLoS One. 2020 Oct 22;15(10):e0240996. doi: 10.1371/journal.pone.0240996 (PMC7580985; doi:10.1371/journal.pone.0240996)
Supplement: Attachment — Submitted filename: Response to Reviewers.docx [file pone.0240996.s008.docx]

**Response to reviewers’ comments (MS PONE-D-20-18642– REV1)**

*Answers to reviewers are reported after each comment*

**Reviewer #1**

**Comment #1** - I believe this manuscript addresses an important question, and that the authors have done what they can with very limited numbers of studies available for analysis. I am left wondering if 5 published studies is an adequate sample number, particularly considering that 2 are from very different systems to the other three, to draw broad conclusions. The work seems very preliminary based on such a small sample size.

*Answer*: Thank you! Yes, we agree that our analysis would benefit from a higher number of studies. In principle, a meta-analysis is defined as “*the statistical combination of results from two or more separate studies*”^[1]^. Also, looking at the published literature (references 37-49 in our study), previous meta-analyses focused on a wide range of studies spanning from 2 (Bisanz et al.) to 30 (Duvallet et al.), with several of them in the range between 2 and 9 (Bisanz et al, Walters et al, Krych et al., Jiao et al., Wirbel et al., Rocca et al.). In addition, we took several steps to guarantee that data analysis would consider the heterogeneity between studies into account. Therefore, we believe that the number of studies and the approach we took guarantee a low bias in our results. We acknowledge these limitations in our discussions (L199-201, clean copy).

**Comment #2** - I have attached a PDF file with some minor comments.

*Answer*: We integrated your suggestions in our manuscript, including a brief description of violin plots in the caption of Fig. 1A (L184-185, clean copy).

**Reviewer #2**

**Comment #1** - This paper is not a meta-analysis. It is an analysis of combined data from five studies, but meta-analysis procedures were not used. Unfortunately, I cannot comment on the microbial data methods and analysis in this paper because it is beyond my area of expertise, but for reasons outlined below, I do feel the paper would be better framed as a review + additional analysis, rather than a meta-analysis (which it is not).

*Answer*: We thank the reviewer for this comment; however, we respectfully disagree with their point of view. A meta-analysis is defined as “*the statistical combination of results from two or more separate studies*”^1^. The NIH NCI defines the meta-analysis as “*A process that analyzes data from different studies done about the same subject …*”^[2]^. Most of the published literature that used our approach define their analysis as “meta-analysis” (refs 37-49 in our paper). We acknowledge that using data for single points^[3]^, rather than aggregated data, is quite unusual. Literature is rich of examples where a meta-analytical approach has been used on individual “samples”^[4]^. Therefore, we believe that meta-analysis is the most appropriate term to use in our case.

**Comment #2** - And the conclusions need to be tempered and put in a greater context.

*Answer*: Thanks for this suggestion. We added a sentence to the last paragraph to put our results in a greater context (L259-260, clean copy).

**Comment #3** - The paper makes broad statements such as, “We show biological invasions decrease the diversity of environmental microbiomes,” which I do not feel are at all justified by the analyses.

*Answer*: We included a statement (L199-201, clean copy) where we acknowledge the limits of our analysis.

**Comment #4** - First, the study includes data from only five papers, three on plants (affecting soil), one on a mammal (affecting soil), and one on an aquatic snail (affecting water). This data set is far too small and disparate to use for sweeping generalizations.

*Answer*: As we mentioned in our answer to comment #1, a meta-analysis can be done with even 2 studies. Furthermore, our analysis considers a total of 335 samples and we took a series of steps (L111-124, clean copy) to alleviate potential sources of bias. We acknowledge the limitations of our study throughout the discussions.

**Comment #5** - Second, it is not at all clear that the results support the statements. For example, a quick look at Figures 2A and 2C would indicate that there are absolutely no differences in either diversity or abundance between control and invaded sites, yet statistical analyses report differences. This makes me question the statistical models used. For example, the mixed model assessing diversity using environment as a random effect is inappropriate because you need more levels (at least 5) for an effect to be random. The unbalanced groups (3 plant, 1 mammal and 1 aquatic snail) could also cause model problems. At the very least, the model diagnostics need to be discussed to show that the model was appropriate.

*Answer*: Thanks for this suggestion. We ran all our models including or excluding “environment” as random effect, and we reported the comparison in the supplementary material. Overall, we found no differences between the two modelling strategies.

**Comment #6** - Finally, I think more needs to be known about individual studies to make any firm conclusions. For example, in study [28] natives were planted in polycultures of up to 7 species and non-natives were planted in monocultures. Does the difference in microbial diversity in such a study represent a realistic shift caused by an invader, or is it a reflection of the experimental design? I think more generalizations could be made if each of the five studies were discussed in some detail and the results put in first a specific context, and then if patters support it, into a more general context.

*Answer*: Thanks for this suggestion. We agree with this comment and we provided a description of the individual studies (L94-106, clean copy). Discussion of our results in the context of individual studies was already provided (L196-201 and L205-215, clean copy).

**Reviewer #3**

**Comment #1** - In the Discussion, the authors explain the limitations and caveats of this study very well. For example, the five studies are mostly about plants, and mostly concern soil microbiomes. Therefore, although I am not refuting the results themselves, I think that some of the conclusions that the authors draw are overly broad. In some places, their claims are well-qualified (line 23: “Our findings suggest that non-native species are responsible…”; line 232: “Without sufficient diversity of sample environments, it is impossible to tell…”), but others seem to overreach. The first line of the Discussion (line 117: “We show biological invasions decrease the diversity of environmental microbiomes.”) feels too broad to me. Although the results did indeed show a decrease in diversity, I think this sentence would benefit from a clause acknowledging that the environmental sampling is limited and biased. The rest of the Discussion does exactly this—pointing out all the ways this sample is biased (soils, invasive plants, northern hemisphere, summer)—so it seems odd to me that this claim is so broad. Alongside these caveats, the Discussion also excellently highlights the overall knowledge gap revealed by this meta-analysis.

*Answer*: Thanks for your suggestion. We included it in the manuscript (L199-201, clean copy).

**Comment #2** - I also thought that the discussion of taxa-level shifts was very interesting. Connecting microbial family shifts to possible shifts in community function is intriguing, although I am very glad that the authors acknowledge that amplicon sequencing cannot definitively show changes in function. In this section, as in the rest of the Discussion, the authors combine the results of their meta-analysis with results of previously published studies smoothly and effectively.

*Answer*: Thank you!

**Comment #3** - Overall, this is an interesting manuscript with a compelling question. However, given the dearth of compatible studies in the final meta-analysis, I think that this manuscript raises many more questions than it answers—this manuscript’s strength is in its ‘call to arms’ for more research rather than in the results themselves.

*Answer*: Thank you!

1. Deeks JJ, Higgins JPT, Altman DG (editors). Chapter 10: Analysing data and undertaking meta-analyses. In: Higgins JPT, Thomas J, Chandler J, Cumpston M, Li T, Page MJ, Welch VA (editors). Cochrane Handbook for Systematic Reviews of Interventions version 6.0 (updated July 2019). Cochrane, 2019. <https://training.cochrane.org/handbook/current/chapter-10>
2. <https://www.cancer.gov/publications/dictionaries/cancer-terms/def/meta-analysis>
3. <https://www.ncbi.nlm.nih.gov/books/NBK305627/?report=classic>
4. Cooper and Patall (10.1037/a0015565), Curran and Hussong (10.1037/a0015914), Lyman and Kuderer (10.1186/1471-2288-5-14), Riley et al. (10.1136/bmj.c221 and 10.1002/sim.3165), Simmonds et al. (10.1191/1740774505cn087oa), Stweart et al. (10.1177/0163278702025001006).
