# Supplementary material for: Biological invasions alter environmental microbiomes: A meta-analysis
Source: PLoS One. 2020 Oct 22;15(10):e0240996. doi: 10.1371/journal.pone.0240996 (PMC7580985; doi:10.1371/journal.pone.0240996)
Supplement: Attachment — Submitted filename: Response to Reviewers.docx [file pone.0240996.s009.docx]

**Response to reviewers’ comments (MS PONE-D-20-18642– REV2)**

*Answers to reviewers are reported after each comment*

**Reviewer #1**

**Comment #1** - I was concerned with the response to reviewer #2, who commented on the statistical analyses. I do agree that many of the comparisons declared statistically different certainly do not appear to be, from the data shown in the figure. E.g. Fig 1c, it seems very unlikely that Sphingomonadaceae were different in control vs invaded – the medians are essentially identical; yet Gemmatimonadaceae are not different? There are a number of such instances in the figures, and the authors need to explain this clearly. I suspect the asterisks indicating significance are incorrectly annotated, maybe? Their assertion that including as a random effect in their analysis and finding it made no difference to the conclusions, does not go a long way to explaining the apparent lack of differences shown in the figures, despite the pairs being labelled as significantly different. Fig 1a likewise seems to show two very similar data distributions, with the medians essentially the same.

*Answer*: We agree with the reviewer that our graphs do not properly show the differences we claim in our text. Thus, we changed our Fig 1: panel A has been moved to the text (L167-168), panel B is now the main figure, and panel C has been moved to S5 table. Furthermore, we integrated our discussion to reflect your comments (L200-203 and L217-218).
